# Paediatric tuberculosis diagnosis using *Mycobacterium tuberculosis* real-time polymerase chain reaction assay: protocol for systematic review and meta-analysis

**DOI:** 10.1186/s13643-019-1137-y

**Published:** 2019-08-30

**Authors:** Emmanuel O. Babafemi, Benny P. Cherian, Betty Ouma, Gilbert Mogoko

**Affiliations:** 1 Microbiology Department, Paediatrics Laboratory Medicine, Birmingham Women’s and Children’s NHS Foundation Trust, Birmingham, UK; 2 Barts Health NHS Trust, Room MICR324, 3rd floor Pathology & Pharmacy Building, 80 Newark Street, London, E1 2ES UK; 3 Chelsea & Westminster Hospital, 369 Fulham Road, London, Greater London SW10 9NH UK; 4 Microbiology Department, IPP Pathology First, Dobson House, Bentalls, Basildon, SS14 3BY UK

**Keywords:** Paediatric, Tuberculosis, *Mycobacterium tuberculosis*, Systematic review, Meta-analysis

## Abstract

**Background:**

Tuberculosis (TB) diagnosis in children is a major challenge with up to 94% of children with TB treated empirically in TB high-burden countries. Paediatric tuberculosis (PTB) remains a major cause of morbidity and mortality globally, particularly in developing countries. Most deaths/morbidity from TB in paediatrics could be prevented with early diagnosis and appropriate treatment.

The main objective of this systematic review is to examine the evidence whether real-time polymerase chain reaction assay could be the most accurate clinical laboratory diagnostic methodology for the *Mycobacterium tuberculosis* (MTB) detection in paediatrics.

**Methods:**

We will search MEDLINE/PubMed, EMBASE, BIOSIS, LILACS, Cochrane Infectious Diseases Group Specialised Register (CIDG SR), Global Health, and CINAHL for published studies that recruited children less than 16 years of age being investigated for *Mycobacterium tuberculosis* (MTB) infection using real-time polymerase chain reaction assay accompanied by mycobacteriological culture investigation as the reference standard. There will be no restriction regarding the language, date of publication, and publication status. We will include randomised controlled trials and observational studies (cohort, cross-sectional) in the review.

Selection of studies, data extraction and management, assessment of risk of bias, and quality of evidence will be performed by two independent reviewers (EB and BC). A third researcher will be consulted in case of discrepancies. Depending on the availability and quality of the data, a meta-analysis will be performed. Otherwise, findings will be qualitatively reported.

**Discussion:**

To our knowledge, this is the first systematic review and meta-analysis assessing the detection of MTB from all clinical sample types using real-time polymerase chain reaction assay in paediatric population. This review will make available evidence on the accuracy, approach, and interpretation of results of this assay in the context of MTB diagnosis which will meet an urgent need, considering the challenges of MTB diagnosis in paediatrics.

**Systematic review registration:**

PROSPERO CRD42018104052

**Electronic supplementary material:**

The online version of this article (10.1186/s13643-019-1137-y) contains supplementary material, which is available to authorized users.

## Background

Tuberculosis (TB) diagnosis in children is a major challenge with up to 94% of children with TB treated empirically in TB high-burden countries. Diagnosis of pulmonary tuberculosis in children has relied predominantly on clinical, radiological, and tuberculin skin test. TB in children is often missed or overlooked due to non-specific symptoms and or non-specific diagnostic tests [1, 2]. TB is an infectious disease caused by the bacillus *Mycobacterium tuberculosis*. It typically affects the lungs (pulmonary TB) but can also affect other sites (extrapulmonary TB). The disease is spread when people who are sick with pulmonary TB expel bacteria into the air, for example by coughing [2].

At least one million children become ill with tuberculosis (TB) each year. Children represent about 10–11% of all TB cases. It is rarely bacteriologically confirmed [3]. Pulmonary TB is one of the top ten killers of children and infants worldwide. Young children are at particular risk of developing severe, often fatal, or lifelong disabling forms of TB. In 2017, 233,000 children died of TB, among whom 52,000 were living with HIV [4, 5].

Paediatric tuberculosis (PTB) remains a major cause of morbidity and mortality globally, particularly in developing countries.

Most deaths from PTB could be prevented with early diagnosis and appropriate treatment [6]. Generally, the lack of a simple and effective diagnostic test that can be utilised in resource-limited settings, where the infection is endemic, has hindered its control [7].

The actual burden of TB in children is likely higher given the challenge in diagnosing childhood TB in many low-income countries where the diagnosis of paediatric TB is solely based on clinical evidence and smear microscopy [8].

Latent tuberculosis infection (LTBI) is defined as a state of persistent immune response to stimulation by MTB antigens with no evidence of clinically manifest active TB [9]. The vast majority have no signs or symptoms of TB disease and are not infectious. Not all individuals infected with MTB develop active TB. It is estimated that the lifetime risk of an individual with LTBI for progression to active TB disease is 5–10% over their lifetime [10]. The risk for active TB disease after infection depends on several factors, the most important being immunological status. This risk is particularly high among children under the age of 5 years [11].

Tuberculin skin test (TST) or interferon-gamma release assay (IGRA) can be used to test for LTBI, as there is no ‘gold standard’ test for LTBI [12]. It is only a marker of exposure, not disease [1].

Establishing an accurate diagnosis of PTB in children can be more difficult than adult TB, because of the challenge children have in expectorating good-quality sputum or absence of lung parenchymal disease as in primary complex.

This leads to a compromise of the quality of sputum smear microscopy results, with the added difficulty that the disease can be paucibacillary, with fewer organisms present in specimens [13]. Culture systems which improve diagnosis take between 2–8 weeks in most cases [14, 15].

Other diagnostic approaches are based on clinical presentations, radiographic abnormalities, contact history, and tuberculin skin test, all of which are of low specificity [16].

The development of real-time polymerase chain reaction (RT-PCR)-based assays for the detection of *Mycobacterium tuberculosis* target genes (DNA) in clinical specimens has proved to be rapid and accurate. Since 2013, molecular tests have also been recommended for use in children and to diagnose specific forms of extrapulmonary TB. The assay has much better accuracy than sputum smear microscopy [17].

This main objective of this systematic review is to synthesis the summary estimates whether RT-PCR assays will be more rapid, sensitive, and specific for diagnosing MTB infection in paediatrics with tuberculosis compared to the culture-based assays.

The outputs of this systematic review will serve as a resource for decision-makers, providing government stakeholders and healthcare practitioners with the tools to make evidence-based decisions for PTB diagnosis and control.

### Research question

Can real-time polymerase chain reaction (RT-PCR) assay be more rapid, sensitive, and specific for routine detection of MTB from paediatric samples in clinical microbiology laboratories compared to the culture-based assays (gold standard)?

In answering this question, our study will address the following framework: Population, Index test, Comparison (reference test), and Outcome (PICO) question.

## Methods

This systematic review protocol has been developed based on the Preferred Reporting Items for Systematic Reviews and Meta-Analyses Protocols (PRISMA-P) guidelines [18], which is available in Additional file 1. The systematic review protocol was registered with the International Prospective Register of Systematic Reviews (PROSPERO) database (registration ID: CRD42018104052).

We will search MEDLINE/PubMed, EMBASE, BIOSIS, LILACS, Cochrane Infectious Diseases Group Specialised Register (CIDG SR), Global Health, and CINAHL using the search strategy and terms used for one of the databases as detailed in Additional file 2. This will be used for published studies that recruited children less than 16 years of age being investigated for *Mycobacterium tuberculosis* (MTB) infection using real-time polymerase chain reaction assay accompanied by mycobacteriological culture investigation as the reference standard. There will be no restriction regarding the language, date of publication, and publication status. The search strategy for each database will be validated by a librarian information specialist familiar with the topic. The electronic search will be tailored for each database to include its specific keywords and MeSH terms.

### Searching other resources

To avoid missing relevant studies to be included, we will search other sources by looking through reference lists of relevant reviews and selected studies, searching websites of a relevant organisation, and searching of relevant articles using the PubMed-related article feature, Google Scholar, Cochrane Library, turning research into practice (TRIP), and portal of the WHO International Clinical Trials Registry Platform (www.who.int/trialsearch) to identify ongoing trials, as well as StopTB Partnership’s New Diagnostics Working Group (www.stoptb.org/wg/new_diagnostics/), the World Health Organization and Centers for Disease Control and Prevention websites, and proceedings of the International Union Against Tuberculosis and Lung disease (UNION) conference. We will also contact leading researchers at the Foundation for Innovative New Diagnostics (FIND). A search of grey literature including conference proceedings (Conference Proceedings Citation Index–Science (CPCI-S)), Dissertations & Theses (www.proquest.com), and expert information will be done and added to our resource material.

### Data collection and analysis

#### Study selection and data extraction

After the literature search, two review authors (EB and BC) will independently screen studies for eligibility. Following screening, selection of studies irrespective of their design provided they meet the inclusion criteria will be carried out by two authors (EB and BC). They will independently review titles and abstracts against eligibility criteria to categorise as either ‘potentially include’ or ‘exclude’ (see Additional file 3, which is the flow chart diagram). A third researcher (BO or GM) will be consulted in case of discrepancies at each of the stages. We will resolve differences in opinion through discussion. We will list studies excluded after full-text assessment and their reasons for exclusion in a ‘Characteristics of excluded studies’ table.

Data will be extracted independently by EB and BC from each selected study using a predetermined list of categories/characteristics: first author, year of publication, participants/population, index test, reference test, country, disease and target sequence gene for MTB DNA detection, and results into a standardised data extraction form (see Additional file 4 Part A)

We will conduct a risk of bias assessment at the level of the study using QUADAS-2 (University of Bristol) tool that assesses diagnostic evaluation work in four domains: (1) patient selection, (2) the index test, (3) the reference standard, and (4) patient flow and timing of tests (see Additional file 4 Part B). We will perform a narrative synthesis, and depending on the availability and quality of the data, a meta-analysis addressing our outcomes will be performed. For studies with missing or incomplete information for meta-analysis, we will contact the authors by using their contact information provided in the studies. When attempts to contact the authors have not been successful, such studies will be excluded from the meta-analysis.

We will utilise the Review Manager (RevMan V5.3, Cochrane Collaboration, Oxford, UK) and Meta-DiSC (version 1.4) statistical software to carry out the meta-analysis [19, 20]. For meta-analysis, if there are enough studies, the bivariate model will be used because it takes into account potential threshold effects and the correlation between sensitivity and specificity. In addition, it will allow addition of covariates for investigation of potential sources of heterogeneity

We will also report point estimates and 95% confidence intervals, for sensitivity and specificity for each study as paired forest plots, and a plot summary receiver operating characteristics (SROC) curve [21, 22], as different thresholds are expected to be used by manufacturers of RT-PCR assays.

### Subgroup analyses

If a meta-analysis is carried out, subgroup analyses will be performed using the following a priori:

1. Index test types and their respective target sequence genes. We will assess performance of different types of RT-PCR assays used for the detection of MTB from all the clinical specimen types and their respective target sequence genes.
2. Type of reference test. The goal of a reference standard test is to provide error-free classification of the disease outcome presence or absence. We will assess the performance of mycobacteriology culture-based approaches in the following regions: (1) studies using solid medium, (2) studies using liquid medium, and (3) studies combining both media.
3. TB classification. We will perform among the participants those who are having pulmonary tuberculosis versus those with extrapulmonary tuberculosis.
4. Impact of RT-PCR assays on low- and middle-income country (LMIC) versus high-income country (HIC). We will assess sources of data to these graders.

### Quality assessment

Two review authors (EB and BC) will independently conduct a risk of bias assessment at the level of the study using the QUADAS-2 (University of Bristol), the recommended tool for evaluating primary studies for the inclusion in systematic reviews for diagnostic accuracy.

QUADAS-2 tool with assessment based on risk of bias and applicability of results has four domains evaluating (1) patient selection, (2) the index test, (3) the reference standard, and (4) patient flow and timing of tests (see Additional file 4 Part B)

### Assessment for heterogeneity and publication bias

We will assess the extent of heterogeneity among studies visually with forest plots and SROC curves with 95% prediction regions and statistically with chi-squared (*χ*^2^) and *I*-squared (*I*^2^) [21, 22]. The source of heterogeneity will be investigated using stratified (subgroup) analyses.

Every effort will be made to identify unpublished studies through searching conference abstracts, grey literature, and reference lists of relevant primary articles to minimise publication bias. Formal assessment of publication bias using methods such as funnel plots or regression tests was not evaluated as this is not usually recommended in the meta-analysis for diagnostic test accuracy [21, 22].

## Discussion

To our knowledge, this is the first systematic review and meta-analysis assessing the paediatric detection of MTB from all clinical sample types using real-time polymerase chain reaction assay. Pooling all available evidence on the accuracy, approach, and interpretation of results of this assay in the context of MTB diagnosis will meet an urgent need, considering the challenges of MTB diagnosis in paediatrics. We therefore believe that our findings will have impact on policy and guide clinical laboratory practice to improve paediatric MTB diagnostic approach. The practicality of using RT-PCR assays in a resource-limited setting will be discussed within the technical challenges, cost, reagents, and other logistics.

Strengths and limitations of included studies and this review will be discussed, and recommendations for further research and clinical practice will be provided.

## Additional files

Additional file 1: PRISMA-P 2015 Checklist. (DOCX 33 kb)

Additional file 2: Search Strategy. (DOCX 14 kb)

Additional file 3: Flow Chart diagram. (DOC 60 kb)

Additional file 4: Part A: Data Extraction formfile 4. Part B: QUADAS-2 (Quality assessment of diagnostic accuracy studies-2 tool). (DOCX 24 kb)

## Abbreviations

CPCI-S: Conference Proceedings Citation Index–Science
EMBASE: Excerpta Medica database
GRADE: Grades of Recommendation, Assessment, Development and Evaluation
HIC: High-income country
IGRA: Interferon-gamma release assay
LMIC: Low- and middle-income country
LTBI: Latent tuberculosis infection
MEDLINE: Medical Literature Analysis and Retrieval System Online
MeSH: Medical Subject Headings
PRISMA: Preferred Reporting Items for Systematic Reviews and Meta-Analysis
PRISMA-P: Preferred Reporting Items for Systematic Reviews and Meta-Analysis Protocols
TRIP: Turning research into practice
TST: Tuberculin skin test
WHO ICTRP: WHO International Clinical Trials Registry Platform
