# Supplementary material for: Paediatric tuberculosis diagnosis using Mycobacterium tuberculosis real-time polymerase chain reaction assay: protocol for systematic review and meta-analysis
Source: Syst Rev. 2019 Aug 30;8:225. doi: 10.1186/s13643-019-1137-y (PMC6716920; doi:10.1186/s13643-019-1137-y)
Supplement: Supplementary file 2 — Search Strategy. (DOCX 14 kb) [file 13643_2019_1137_MOESM2_ESM.docx]

**ADDITIONAL FILE 2: Search strategy**

The following Medline via PubMed search algorithm will be translated to EMBASE**:**

1. ("tuberculosis") ti,ab

2. (mycobacterium tuberculosis) ti,ab

3. (extrapulmonary tuberculosis) ti,ab

4. (pulmonary tuberculosis) ti,ab

5. (paediatric tuberculosis) ti,ab

6. 1 OR 2 OR 3 OR 4 OR 5

7. ("Real-time polymerase chain reaction") ti,ab

8. (real-time pcr) ti,ab

9. (real-time pcr assay) ti,ab

10. ("rt-pcr") ti,ab

11. ("Nucleic Acid Amplification Test") ti,ab

12. ("NAAT") ti,ab

13. 7 OR 8 OR 9 OR 10 OR 11 OR 12

14. ("culture-based media") ti,ab

15. (culture-based assay) ti,ab

16. ("liquid media") ti,ab

17. ("solid media") ti,ab

18. 14 OR 15 OR 16 OR 17

19. (“paediatric”) ti,ab

20. (“paediatrics”) ti,ab

21. (“children”) ti,ab

22. 19 OR 20 OR 21

23. 6 AND 13

24. 18 AND 22

25. 23 AND 24
