# Supplementary material for: Paediatric tuberculosis diagnosis using Mycobacterium tuberculosis real-time polymerase chain reaction assay: protocol for systematic review and meta-analysis
Source: Syst Rev. 2019 Aug 30;8:225. doi: 10.1186/s13643-019-1137-y (PMC6716920; doi:10.1186/s13643-019-1137-y)
Supplement: Supplementary file 4 — Part A: Data Extraction formfile 4. Part B: QUADAS-2 (Quality assessment of diagnostic accuracy studies-2 tool). (DOCX 24 kb) [file 13643_2019_1137_MOESM4_ESM.docx]

**ADDITIONAL FILE 4 (Part A): Data extraction form**

| Study ID (first author and year of publication) |
| --- |
|  |
| Title: |

| **Notes:** |
| --- |

1. **General Information**

| **Date form completed**  **(dd/mm/yyyy)** |  |
| --- | --- |
| **Name/ID of person extracting data** |  |
| **Reference citation** |  |
| **Study author contact details** |  |
| Was author contacted? |  |
| **Publication type**  **(e.g. full report, abstract, letter)** |  |
| **Notes:** | |

**B) Study eligibility**

| **Study characteristics** | | **Include** | **Exclude** |
| --- | --- | --- | --- |
| **Study design**  - Other, specify:  - Unknown/not reported If other, specify: | | Cohort  Prospective  Retrospective  Others  Specify: | Design-free  Unknown/not reported |
| **Participants** | | Active tuberculosis  Latent tuberculosis  Others | Other mycobacterium infection |
| **Index test** | | Real-time polymerisation chain reaction | Microscopy  Other test(s) |
| **Reference test** | | Culture-based assay  Solid:  Liquid:  Both solid and liquid culture: | No reference test |
| **Target sequence** | |  |  |
| **Outcomes** | | Sensitivity  Specificity  Rapid  Others | No outcomes |
| **Data** | | Sufficient for 2X2 contigency table | Insufficient data |
| **Other reason for exclusion** | **DO NOT PROCEED IF STUDY IS EXCLUDED FROM REVIEW** | | |
| **Notes:** | | | |

1. **Characteristics of included studies**

| **Aim/Objectives of the study** | **Descriptions as stated in paper** | **Location in text** **or source** (pg, fig/table/other |
| --- | --- | --- |
|  |  |  |
| **Country** (where the study was conducted) | Low-and middle-income country (LMIC) and High-income country (HIC) |  |
| **Study design** (e.g.  Prospective, Retrospective,  Cohort  Cross-sectional,  Unknown/not reported | Cross-sectional, primary observational studies and Randomised controlled trials (RCTs) - blind or unblended- with a high degree of heterogeneity are the only eligible studies. Cohort studies |  |
| **Ethical approval needed/obtained for study** | Yes No Unclear |  |
|  |  |  |
| **Notes:** | | |

1. **Participant’s characteristics**

|  | **Description** | **Comments** |
| --- | --- | --- |
| **Population** |  |  |
| **Age** |  |  |
| **Sex** |  |  |
| **Race/Ethnicity** |  |  |
| **Disease characteristics** |  |  |
| **Specimen types** | Pulmonary  Extra-pulmonary |  |
| **Number of included specimens** | Pulmonary  Extra-pulmonary |  |
| **Notes**: | | |

**ADDITIONAL FILE 4 (Part B):** Quality assessment of diagnostic accuracy studies-2 tool

QUADAS-2 tool: Risk of bias and applicability judgments

| Domain 1: Patient selection | |
| --- | --- |
| 1. Risk of bias |  |
| Describe methods of patient selection: | |
| - Was a consecutive or random sample of patients enrolled? | Yes/No/Unclear |
| - Was a case-control design avoided? | Yes/No/Unclear |
| - Did the study avoid inappropriate exclusions? | Yes/No/Unclear |
| Could the selection of patients have introduced bias? | RISK: LOW/HIGH/UNCLEAR |
| 1. Concerns regarding applicability |  |
| Describe included patients (prior testing, presentation, intended use of index test and setting): | |
| Is there concern that the included patients do not match the review question? | CONCERN: LOW/HIGH/UNCLEAR |
| Domain 2: Index test(s) *(if more than 1 index test was used, please complete for each test)* | |
| 1. Risk of bias |  |
| Describe the index test and how it was conducted and interpreted: | |
| - Were the index test results interpreted without knowledge of the results of the reference standard? | Yes/No/Unclear |
| - If a threshold was used, was it pre-specified? | Yes/No/Unclear |
| Could the conduct or interpretation of the index test have introduced bias? | RISK: LOW/HIGH/UNCLEAR |
| 1. Concerns regarding applicability |  |
| Is there concern that the index test, its conduct, or interpretation differ from the review question? | CONCERN: LOW/HIGH/UNCLEAR |
| Domain 3: Reference standard | |
| 1. Risk of bias |  |
| Describe the reference standard and how it was conducted and interpreted: | |
| - Is the reference standard likely to correctly classify the target condition? | Yes/No/Unclear |
| - Were the reference standard results interpreted without knowledge of the results of the index test? | Yes/No/Unclear |
| Could the reference standard, its conduct, or its interpretation have introduced bias? | RISK: LOW/HIGH/UNCLEAR |
| 1. Concerns regarding applicability |  |
| Is there concern that the target condition as defined by the reference standard does not match the review question? | CONCERN: LOW/HIGH/UNCLEAR |
| Domain 4: Flow and timing | |
| 1. Risk of bias |  |
| Describe any patients who did not receive the index test(s) and/or reference standard or who were excluded from the 2x2 table (refer to flow diagram):  Describe the time interval and any interventions between index test(s) and reference standard: | |
| - Was there an appropriate interval between index test(s) and reference standard? | Yes/No/Unclear |
| - Did all patients receive a reference standard? | Yes/No/Unclear |
| - Did patients receive the same reference standard? | Yes/No/Unclear |
| - Were all patients included in the analysis? | Yes/No/Unclear |
| Could the patient flow have introduced bias? | RISK:  LOW/HIGH/UNCLEAR |
